# Effects of aerobactin-encoding gene *iucB* and regulator of mucoid phenotype *rmpA* on the virulence of *Klebsiella pneumoniae* causing liver abscess

**DOI:** 10.3389/fcimb.2022.968955

**Published:** 2022-11-11

**Authors:** Shixing Liu, Zeyu Huang, Jingchun Kong, Yining Zhao, Mengxin Xu, Beibei Zhou, Xiangkuo Zheng, Dandan Ye, Tieli Zhou, Jianming Cao, Cui Zhou

**Affiliations:** ^1^ Department of Clinical Laboratory, The First Affiliated Hospital of Wenzhou Medical University; Key Laboratory of Clinical Laboratory Diagnosis and Translational Research of Zhejiang Province, Wenzhou, China; ^2^ Department of Clinical Laboratory, The Children’s Hospital of Zhejiang University School of Medicine, Hangzhou, China; ^3^ School of Laboratory Medicine and Life Science, Wenzhou Medical University, Wenzhou, Zhejiang, China

**Keywords:** *Klebsiella pneumoniae*, liver abscess, aerobactin, mucoid phenotypic regulatory gene *rmpA*, virulence

## Abstract

This study aimed to analyze the influence of the main aerobactin-encoding gene *iucB* and the regulator of mucoid phenotype *rmpA* on the virulence of *Klebsiella pneumoniae* causing liver abscess. In addition, the possible regulatory effects of the main encoding gene *iucB* on the regulator of mucoid phenotype *rmpA* were explored, thus providing novel strategies for the prevention and control of hypervirulent *K. pneumoniae* (hvKp) causing liver abscess. The virulence-related genes *iucB* and *rmpA* of *K. pneumoniae* were detected by PCR. *iucB* and *rmpA* were cloned into *K. pneumoniae* strain by using plasmid pET28b as vector. Quantitative real-time PCR (RT-qPCR) was employed to detect the relative expression of *rmpA* gene in *K. pneumoniae*. We investigated the potential effects of aerobactin coding gene *iucB* and regulator of mucoid phenotype *rmpA* on the virulence of *K. pneumoniae* by establishing the *Galleria mellonella* infection model. Capsule quantitative experiment was conducted to investigate the impact of aerobactin-encoding gene *iucB* on the modulation of regulator of mucoid phenotype *rmpA*. The results of the *G. mellonella* infection model indicated that *iucB* gene could significantly enhance the virulence of *K. pneumoniae*, but the presence of *rmpA* gene did not markedly affect the virulence of *K. pneumoniae*. RT-qPCR showed that *iucB* inhibited the expression of *rmpA* gene. Quantitative capsulation experiments showed that the presence of *rmpA* gene could not increase the capsulation production of *K. pneumoniae*. The main encoding gene of aerobactin, namely *iucB*, could substantially enhance the virulence of *K. pneumoniae*. The gene *iucB* might be involved in the biosynthesis of the capsular polysaccharide through an unknown mechanism instead of the gene *rmpA*. Overall, these findings provide important theoretical support for the treatment of infections caused by hvKp.

## Introduction

*Klebsiella pneumoniae*, as a member of *Enterobacteriaceae*, is an important pathogen causing community and nosocomial-acquired infection. At present, it has emerged as the second-largest opportunistic pathogen after *Escherichia coli* and could cause pneumonia, urinary tract infection, bacteremia, meningitis, as well other infections in individuals with low immunity (Paczosa and Mecsas, 2016). It has been also found that, hypervirulent *K. pneumoniae* (hvKp) can also cause highly invasive infections in the healthy young individuals, such as liver abscess, and can spread from the original infection site to other organs. Once severe invasive spread occurs, patients are often affected with serious and irreversible refractory sequelae, such as blindness and central nervous system damage (Shon et al., 2013; Prokesch et al., 2016). Therefore, there is an urgent need to understand the different virulence factors and potential effects of virulence-related genes in *K. pneumoniae*. This can lead to the development of personalized and precise treatment measures to effectively treat liver abscess caused by *K. pneumoniae*.

The main virulence factors of *K. pneumoniae* are capsule, lipopolysaccharide, fimbriae, and siderophores (Podschun and Ullmann, 1998). The latest findings indicate that the regulator of mucoid phenotype *rmpA* can significantly increase the production of the bacterial capsules (Cheng et al., 2010). At the same time, a number of prior research studies have shown that compared with non-high virulence *K. pneumoniae*, the carrier rate of gene *rmpA* in hvKp strains was close to 100% (Surgers et al., 2016; Wu et al., 2017; Yu et al., 2017), which suggested that *rmpA* has a significant correlation with hvKp. These hvKp strains expressed *rmpA*, and at the transcriptional level up-regulated the *cps* gene cluster required for *K. pneumoniae* synthesis of capsules, thus resulting in the overproduction of capsules. Thus, it was more robust and easier to colonize at the site of disease than the typical capsules. The stronger is the tendency to colonize, the greater could be the threat to the host. Siderophore is another important virulence factor secreted by *K. pneumoniae*, which can enable the bacteria to obtain iron by transporting ferric ions into the cell, thereby promoting the growth and metabolism of bacteria and thus aggravating the infection and diffusion of bacteria. Siderophores include aerobactin, enterobactin, salmonocin, and yersinomycin. Among them, aerobactin has been closely related to the invasive infection, thus accounting for more than 90% of active siderophore (Russo et al., 2014), and is the main siderophore system of *K. pneumoniae* the gene *iucABCDiutA* encoded aerobactin, of which *iucB* was identified as the most important encoding gene of aerobactin.

In addition, some previous studies have indicated that aerobactin primarily transports iron from the host tissue cells, and could increase the content of siderophore in *K. pneumoniae* even under iron-poor conditions [9], which was identified as the most important virulence factor in siderophores of *K. pneumoniae* (Li et al., 2019). Russo et al. also proved that aerobactin played an important role to increase the virulence of *K. pneumoniae* through experiments *in vitro/vivo* (Russo et al., 2015). In the presence of iron uptake regulators that can reduce iron uptake and iron storage to maintain iron balance, the expression of *rmpA* was significantly inhibited. Moreover, the expression of *rmpA* was significantly upregulated by constructing a mutant with iron uptake regulator deletion (Cheng et al., 2010). Therefore, starting from the regulatory site of the bacterial intracellular iron content, exploring the regulatory effect of Aerobactin encoding gene *iucB* on the main virulence factor *rmpA* of *K. pneumoniae* might be an important strategy for developing novel clinical anti-infection therapies.

## Materials and methods

### Plasmids, bacterial strains, and growth conditions

The various bacterial strains and plasmids used in the present study have been listed in **Table 1**. Among them, the different bacterial strains were recovered from the First Affiliated Hospital of Wenzhou Medical University in China. *K. pneumoniae* FK3973 and its derivatives were propagated at 37°C in Luria-Bertani (LB) broth. The antibiotic used was kanamycin (50 μg/ml). The isolates were stored in LB (Luria-Bertani) medium containing 30% glycerol at –80°C for the further analysis. All investigation protocols in this study have been approved by the ethics committee of the First Affiliated Hospital of Wenzhou Medical University. Informed consent was waived because this study with observational nature focused mainly on the bacteria and involved no interventions for the patients.

**Table 1:** Plasmids and bacterial strains used in this study.

Plasmid or strains	Description	Source
Plasmid		
pET28b	Expression plasmid carrying kanamycin resistance gene	This study
pET28b::*iucB*	The gene *iucB* linked to the plasmid pET28b	This study
pET28b::*rmpA*	The gene *rmpA* linked to the plasmid pET28b	This study
pET28b::*iucB*::*rmpA*	The gene *iucB* and *rmpA* linked to the plasmid pET28b	This study
Strains		
*K. pneumoniae* FK3973	Carrying the genes *iucB* and *rmpA*	Isolated from liver abscess
*K. pneumoniae* FK2931	No carrying the genes *iucB* and *rmpA*	Isolated from liver abscess
pET28b FK2931	FK3931 introduced into the plasmid pET28b	This study
pET28b::*iucB* FK2931	FK3931 introduced into the plasmid pET28b::*iucB*	This study
pET28b::*rmpA* FK2931	FK3931 introduced into the plasmid pET28b::*rmpA*	This study
pET28b::*iucB*::*rmpA* FK2931	FK3931 introduced into the plasmid pET28b::*iucB*::*rmpA*	This study

### PCR assay and cloning

The genome DNA of the two different strains FK3973 and FK2931 was extracted using the Biospin Bacterial Genomic DNA Extraction kit (Bioflux, Tokyo, Japan) according to the manufacturer’s instructions and served as templates for the subsequent analysis. *iucB* and *rmpA* were identified by PCR. The primers of all genes for PCR are listed in **Table S1**. The products of PCR amplification were sequenced by Shanghai Genomics Institute Technology Co. Ltd (Shanghai, China), and the sequencing data was analyzed using BLAST search against the NCBI database (www.ncbi.nlm.nih.gov/BLAST).

As for *iucB* cloning, the pET28b plasmid and *iucB* PCR products were digested with NotI and SalI-restriction enzyme and incubated overnight. The complementation plasmid was transformed into *E. coli* DH5α and screened on LB agar plates containing 50 μg/mL of kanamycin at 37°C overnight. The plasmid DNA was extracted and electroporation into the competent cells of the FK2931 and then screened on LB plates supplemented with 50 μg/mL of kanamycin. The plasmid was extracted for PCR (*iucB* (SalI)F and *iucB* (NotI)R), and the positive-amplification products were confirmed by sequencing. As for *rmpA* cloning, the pET28b plasmid and *rmpA* PCR products were digested with BamHI and SacI-restriction enzyme and then connected overnight. The rest of the procedure was similar to *iucB* cloning. Finally, the plasmid was extracted for PCR (*rmpA*(BamHI)F and *rmpA*(SacI)R), whereas the positive-amplification products were confirmed by sequencing. As for co-cloning of *iucB* and *rmpA*, the pET28b::*iucB* plasmid was constructed as above and *rmpA* PCR products were digested with BamHI and SacI-restriction enzyme and connected overnight. The rest of the procedure was similar to rmpA cloning. For the pET28b empty vector strains, the pET28b plasmid was electroporated into the competent cells of FK2931 and thereafter screened on LB plates supplemented with 50 μg/mL of kanamycin.

### Extraction and quantification of the capsule

Uronic acid was extracted and quantified as described previously (Yang et al., 2019). Briefly, 500 μL of the bacterial culture were grown in LB broth for 6 h and then tested in the microviscosity assay were mixed with 100 μL of 1% ZWITTERGENT 3-14 detergent in 100 mM citric acid, followed by incubation at 50°C for 20 min. The cells were then pelleted and 300 μL of the supernatant were added to 1.2 mL of absolute ethanol, incubated at 4°C for 20 min, and then centrifuged for 5 min at maximum speed. The pellet was dried and resuspended in 200 μL of distilled water, to which 1.2 mL of 12.5 mM sodium tetraborate in sulfuric acid was added and incubated for 5 min at 100°C, and then incubated on ice for 10 min. A 20-μL volume of 0.15% 3-phenyl phenol in 0.5% NaOH was thereafter added. After a 5-min incubation at the room temperature, the absorbance at 520 nm was measured. The glucuronic acid content was then determined from a standard curve of glucuronic acid and expressed as μg OD unit-1. The results have been presented as the mean and s.d. of the data of three independent experiments. An unpaired two-sided Student’s *t*-test was performed to analyze the statistical difference between the viscosity level of parental strains and the transconjugants carrying the hypervirulence plasmid. Statistical analysis was performed by using Graphpad Prism 8.0.

### Quantitative real-time PCR

The total RNA of strains pET28b::*rmpA* FK2931 and pET28b::*iucB*::*rmpA* FK2931 were extracted. 500 ng RNA was then mixed with the reverse transcription system, and 10 μL of cDNA was obtained using a PrimeScript™ RT Kit (TaKaRa, Japan). Thereafter, by using a CFX-96 touch real-time PCR system, qPCR (Bio-Rad, CA, USA) was performed. After this step,100 ng cDNA, TB Green Premix Ex Taq II (Tli RNaseH Plus) (2×) (TaKaRa), and the specific primers (*rmpA*(BamHI) F: 5’-CGGGATCCTACCGTGATTGATTGAATTTT-3’, *rmpA*(SacI) R: 5’-CGAGCTCTTACCTAAATACTTGGCATGAGC-3’) were added to each sample. The cycling conditions used were as follows: 95°C for 30 s, followed by 40 cycles of 95°C for 5 s and 60°C for 30 s. The expression levels of gene *rmpA* were detected by RT-qPCR; *16S rRNA* gene was used as the internal gene. In addition, compared with pET28b::*rmpA* FK2931, the target strain pET28b::*iucB*::*rmpA* FK2931 was quantified using the comparative threshold cycle 2^-ΔΔCt^ method. All experiments were repeated three times independently and average data was used for the calculation of relative expression levels.

### Evaluation of virulence *in vivo* in the infection model of *G. mellonella*

The individual bacterial colony (of FK3973, FK2931, pET28b FK2931, pET28b::*iucB* FK2931, pET28b::*rmpA* FK2931, and pET28b::*iucB*::*rmpA* FK2931) grown overnight was diluted to a 0.5 McFarland standard in normal saline (NS). Thereafter, insects weighing 250–350 mg were selected for the experiment, and those injected with NS were used as the negative controls. Briefly, for the comparison of the virulence of FK2931, pET28b FK2931, and pET28b::*iucB* FK2931, 10 µL of the 0.5 McFarland bacterial solution was injected into the rear left proleg of *G. mellonella* using a microinjector and incubated at 37°C. As for the comparison of virulence of all the selected strains, after the 0.5 McFarland bacteria solution was diluted tenfold, we used 10 µL for injection. Subsequently, the survival rate of *G. mellonella* was recorded after 1, 2, 3, 4, 5, 6, and 7 days. Mortality rates and pathological changes in the vital organs were assessed by Kaplan–Meier analysis and log-rank test. *Larvae* were considered dead when they repeatedly failed to respond to the physical stimuli. All experiments were conducted in triplicate.

### Statistical analysis

All the data was analyzed using the GraphPad Prism v8.0.1 statistical software package (GraphPad Software, La Jolla, CA, USA). The unpaired Student’s t-test (two-tailed) was employed to compare the production of capsules. Kaplan–Meier analysis and log-rank test were performed to analyze the survival rate of *G. mellonella* larvae. *P*-value < 0.05 was considered as statistically significant.

## Results

### *iucB* enhanced the virulence of *K. pneumoniae*

**Figure 1:**
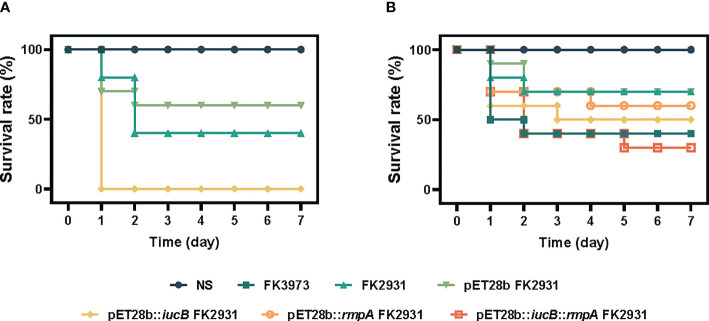
Survival rate of *G. mellonella larvae.* **(A)** *iucB* enhanced the virulence of *K. pneumoniae*. **(B)** Effect of *rmpA* on the virulence of strains.

In this study, by establishing the infection model of *G. mellonella larvae*, we compared the potential virulence differences between the parental strain FK2931, the empty vector strain pET28b FK2931, and the cloned strain pET28b::*iucB* FK2931. As depicted in **Figure 1A**, the virulence of the cloned strain pET28b::*iucB* FK2931 was found significantly stronger in comparison to the parental strain FK2931 and the empty vector strain pET28b FK2931 (*P* <0.05).

### *rmpA* did not affect the capsule of *K. pneumoniae*

**Figure 2:**
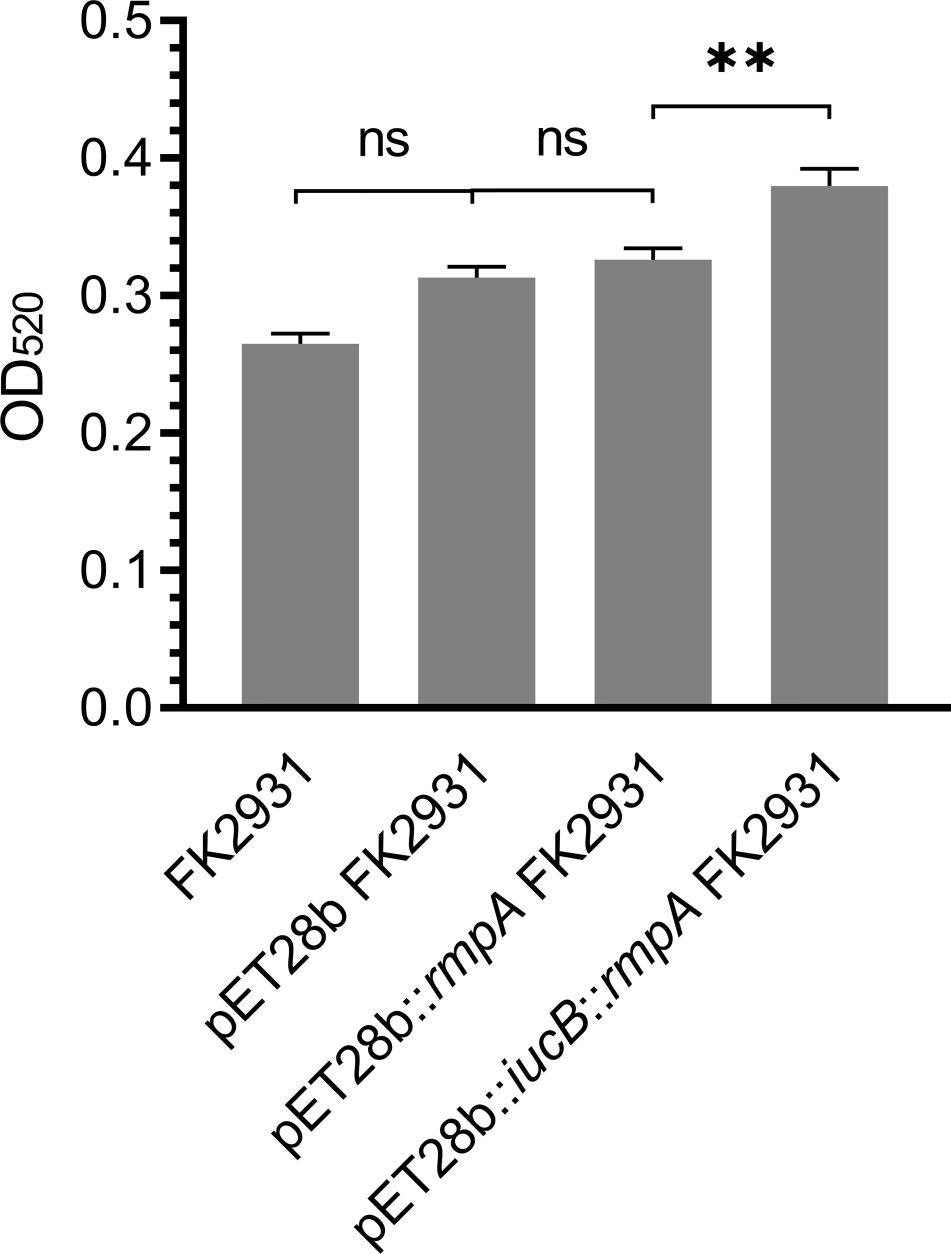
Capsule Quantification Experiment. ** indicates a statistical difference. ns means no statistical difference.

The possible effect of *rmpA* on the capsule content of *K. pneumoniae* has been shown in **Figure 2**. The results indicated that there was no significant differences found in the production of capsules between pET28b FK2931 and pET28b::*rmpA* FK2931. This finding indicated that the presence of *rmpA* could not increase the production of capsules in FK2931 strain; however, the production of capsules in pET28b::*iucB*::*rmpA* FK2931 was significantly higher than that in pET28b::*rmpA* FK2931 (*P <*0.05).

### *iucB* inhibited the expression o*f rmpA*

**Figure 3:**
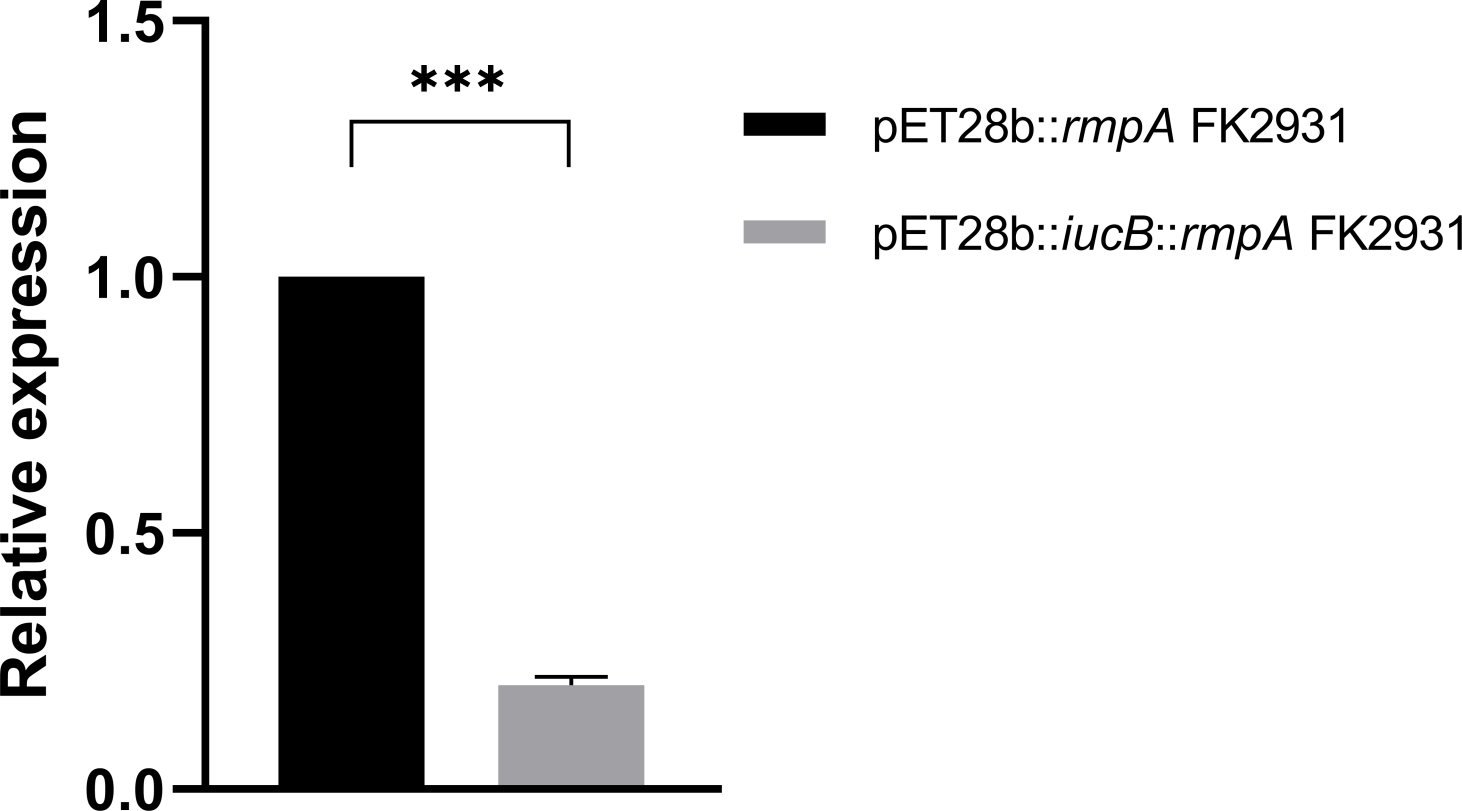
Effect of *iucB* gene on the expression of *rmpA* gene. *** indicates a statistical difference.

As shown in **Figure 3**, the presence of the gene *iucB* significantly inhibited the expression of the gene *rmpA* (*P*<0.05).

### Effect of *rmpA* on the virulence of different strains

We compared the virulence differences among FK3973, FK2931, pET28b FK2931, pET28b::*iucB* FK2931, pET28b::*rmpA* FK2931, and pET28b::*iucB*::*rmpA* FK2931 through establishing an infection a preclinical model of *G. mellonella larvae*. As shown in **Figure 1B**, no significant difference was observed in virulence among FK2931, pET28b FK2931, and pET28b::*rmpA* FK2931 strains (*P*>0.05), which suggested that the presence of *rmpA* did not substantially affect the virulence of *K. pneumoniae*.

## Discussion

In recent years, incidence of *K. pneumoniae* liver abscess (KPLA), an invasive syndrome, has been reported to increase rapidly in Asia and has attracted significant attention due to its high morbidity and mortality. KPLA is usually caused by hvKp, which is highly pathogenic *K. pneumoniae* distinct from classic *K. pneumoniae*. It usually has high viscosity phenotype and could cause life-threatening invasive infections with high pathogenicity and mortality rate in healthy individuals (Lan et al., 2020).

Capsular polysaccharide (CPS), siderophores, lipopolysaccharide (LPS), and fimbriae are the primary factors associated with high virulence in hvKp (Parrott et al., 2021). During infection, *K. pneumoniae* produces siderophores that can chelate iron, markedly decrease transferrin as well as lactoferrin, and generate a soluble iron complex that can be actively transported into the bacteria (Lawlor et al., 2007). In general, most hvKp strains express three types of siderophores: aerobactin, salmonocin, and yersinomycin, encoded by *iucABCDiutA*, *iroBCDN*, and *irp-ybt-fyu*, respectively. A number of studies have demonstrated that aerobactin was the main virulence determinant in hvKp siderophores thus leading to systemic infection. Aerobactin serves as the main factor mediating virulence in the mouse models of intraperitoneal and subcutaneous infection (Russo et al., 2014). It has been found that in more than 90% of hvKp strains, compared with other siderophores, specificity if aerobactin can be used for the discrimination of hvKp, which suggests that it might act as a reliable biomarker of hvKp (Russo et al., 2018). In addition, the genes *iucABCD* and *iutA* are responsible for aerobactin biosynthesis and transport (Bailey et al., 2018). Among them, *iucB* is an important link which is involved in the biological activity of aerobactin. The purpose of our study was to explore the potential effect on the virulence of *K. pneumoniae* by cloning the *iucB* gene. The virulence differences between the cloned strain and the parental strain were compared through establishing an infection model of *G. mellonella larvae*. The results showed that the virulence of the cloned strain pET28b::*iucB* FK2931 was significantly stronger in comparison to the parental strain without the gene *iucB*, thus indicating that the gene *iucB* could markedly enhance the virulence of *K. pneumoniae*, which was consistent with the previous reports that *iucB* can enhance bacterial virulence (Nassif and Sansonetti, 1986). However, the specific mechanisms about how it enhances the virulence of *K. pneumoniae* still needs further exploration.

In hvKp, the capsule was found to be associated with a large virulence plasmid (Lin et al., 2011) that can encode multiple virulence factors, including two plasmid vector transcriptional regulators, the regulators of mucoid phenotype *rmpA* and *rmpA2*. The specific mechanism proposed was that *rmpA* and *rmpA2* bound to the 5’ region of the synthetic gene of the capsule, belonging to the family of transcriptional regulators. Moreover, *rmpA*, 636 bp in size, was first discovered in 1989 and can encode a protein of 137 amino acids that can regulate capsule synthesis, which promoted mucus phenotype formation and enhanced strain virulence. Therefore, in this study, first by employing the cloning experiment, the *rmpA* gene was connected with the plasmids pET28b and pET28b::*iucB* to form the target plasmids pET28b::*rmpA* and pET28b::*iucB*::*rmpA*. Thereafter, finally pET28b::*rmpA* FK2931 and pET28b::*iucB*::*rmpA* FK2931 cloned strains were constructed and was used to investigate the effect of *rmpA* gene on the virulence of *K. pneumoniae* by capsular quantification experiment and setting up infection model of *G. mellonella larvae*. The results indicated that the *rmpA* gene could not enhance the virulence of *K. pneumoniae*, which was contrary to the previous reports. However, some studies have reported that there were three main genes encoding *rmpA/rmpA2*: *rmpA* could be located in both the chromosomes (c-*rmpA*) and plasmid (p-*rmpA*), whereas *rmpA2* was located only in the plasmid (p-*rmpA2*). Moreover, previous study by Cheng et al. showed that KPCG43, p-*rmpA*, and p-*rmpA2* can all promote capsular formation (Cheng et al., 2010). In another report, Hsu et al. compared the NTUH-K2044 strain with the c-*rmpA*, p-*rmpA*, and p-*rmpA2* gene deletion strains, respectively, thus indicating that only p-*rmpA* can significantly enhance the expression of capsular polysaccharide synthesis genes and CPS generation (Hsu et al., 2011). We speculated that the *rmpA* gene in this study could be c-*rmpA*, which failed to enhance capsular polysaccharide synthesis. Therefore, the role of the gene *rmpA* in the formation of virulence still needs to be confirmed in future studies. Although 92%~100% of hvKp carried *rmpA* (Lee et al., 2017), the possible link between *rmpA* and the hypermucus phenotype was not absolute, but some strains with *rmpA* lack the hypermucus phenotype and display low virulence, which may be caused by the simultaneous mutation of the genes *rmpA* and *rmpA2* in the absence of c-*rmpA.*

In this study, we found that the expression of *rmpA* in pET28b::*iucB*::*rmpA* FK2931 was significantly lower than that in pET28b::*rmpA* FK2931, thereby indicating that the presence of *iucB* interfered with the expression of *rmpA*, but the mechanism remained unclear which required further exploration.

In addition, we found that the cloned strain pET28b::*iucB*::*rmpA* FK2931 could significantly produce more capsules than the pET28b::*rmpA* FK2931 strain, but the *rmpA* in this study might be located on the chromosome, which did not promote the capsule formation. Moreover, the presence of the gene *iucB* inhibited the expression of the gene *rmpA*, thus indicating that *iucB* might be involved in promoting the synthesis of capsular polysaccharide through an unidentified mechanism, rather than regulating capsule formation through *rmpA*.

## Conclusion

In conclusion, our study showed for the first time that the main encoding gene *iucB* of aerobactin did not significantly promote the expression of the regulators of mucoid phenotype *rmpA*. The findings emphasized that *iucB* might be involved in the synthesis of the capsular polysaccharide, which can provide important theoretical basis for developing novel treatment modalities against infections caused by hvKp.

## Data availability statement

The original contributions presented in the study are included in the article/**Supplementary Material**. Further inquiries can be directed to the corresponding authors.

## Author contributions

SL conducted the experiments, analyzed the data, and wrote the manuscript. ZH participated in experiments and writing. JK and YZ provided isolates and analyzed the data. MX and BZ participated in the analysis of the results. XZ and DY revised the manuscript. TZ, JC, and CZ helped design the study. All authors read and approved the manuscript.

## Funding

This work was supported by research grants from the National Natural Science Foundation of China (no. 82072347) and the Health Department of Zhejiang Province of the People’s Republic of China (no. 2019KY098).

## Conflict of interest

The authors declare that the research was conducted in the absence any commercial or financial relationships that could be construed as a potential conflict of interest.

## Publisher’s note

All claims expressed in this article are solely those of the authors and do not necessarily represent those of their affiliated organizations, or those of the publisher, the editors and the reviewers. Any product that may be evaluated in this article, or claim that may be made by its manufacturer, is not guaranteed or endorsed by the publisher.
