# Supplementary material for: Effects of aerobactin-encoding gene iucB and regulator of mucoid phenotype rmpA on the virulence of Klebsiella pneumoniae causing liver abscess
Source: Front Cell Infect Microbiol. 2022 Nov 11;12:968955. doi: 10.3389/fcimb.2022.968955 (PMC9691756; doi:10.3389/fcimb.2022.968955)
Supplement: Supplementary file 1 [file Table_1.docx]

**TABLE S1** Primer sequence, production size and annealing temperature used in this study.

| **Primer** | **Suquence** | **Size (bp)** | **Annealing (℃)** |
| --- | --- | --- | --- |
| *iucB* | *iucB*(SalI)F：  ACGCGTCGACATGTCTAAGGCAAACATCGTTCACA  *iucB*(NotI)R：AAGGAAAAAAGCGGCCGCTTACAGACCGACCTCCGTGAAG | 974 | 63 |
| *rmpA* | *rmpA*(BamHI)F ：  CGGGATCCTACCGTGATTGATTGAATTTT  *rmpA*(SacI)R：CGAGCTCTTACCTAAATACTTGGCATGAGC | 791 | 61 |
